# On Fusing Wireless Fingerprints with Pedestrian Dead Reckoning to Improve Indoor Localization Accuracy

**DOI:** 10.3390/s25051294

**Published:** 2025-02-20

**Authors:** Gimo C. Fernando, Tinghao Qi, Edmund V. Ndimbo, Assefa Tesfay Abraha, Bang Wang

**Affiliations:** 1 School of Information, Electronic and Communications, Huazhong University of Science and Technology, Wuhan 430074, China; i202321150@hust.edu.cn (G.C.F.); i202321163@hust.edu.cn (E.V.N.); 2 Mekelle Institute of Technology, Mekelle University, Mekelle 7000, Ethiopia; 3 Hubei Key Laboratory of Smart Internet Technology, Huazhong University of Science and Technology, Wuhan 430074, China

**Keywords:** indoor positioning, fusion algorithm, inertial sensors, signal fingerprints, pedestrian dead reckoning, multi-source data fusion

## Abstract

Accurate indoor positioning remains a critical challenge due to the limitations of single-source systems, such as signal instability and environmental obstructions. This study introduces a multi-source fusion positioning algorithm that integrates inertial sensors and signal fingerprints to address these issues. Using a weighted fusion method, the algorithm employs pedestrian dead reckoning (PDR) for trajectory tracking and combines its outputs with wireless signal fingerprints. Experimental evaluations conducted on diverse trajectories reveal significant improvements in accuracy, achieving a 35.3% enhancement over wireless-only systems and a 71.4% improvement compared to standalone PDR. The proposed method effectively balances computational efficiency and accuracy, demonstrating robustness in complex and dynamic indoor environments. These findings establish the algorithm’s potential for practical applications in navigation, robotics, and Industry 4.0, where precise indoor localization is essential.

## 1. Introduction

Indoor positioning systems (IPSs) have become critical components in various applications, including navigation, robotics, and Industry 4.0 environments [1]. Despite their growing importance, achieving high accuracy in indoor localization remains a significant challenge due to environmental factors such as signal obstructions, multipath effects, and dynamic conditions. Traditional single-source positioning methods, such as Wi-Fi or Bluetooth-based systems, often exhibit limited reliability in complex environments owing to their susceptibility to environmental inconsistencies [2,3,4].

To deal with these problems, researchers are increasingly looking into multi-source data fusion techniques. These techniques combine data from different types of sensors, such as inertial sensors, ultra-wideband (UWB) signals, and geomagnetic signals. These approaches enhance localization performance by leveraging the complementary strengths of diverse modalities [5,6,7]. Among these, pedestrian dead reckoning (PDR), which relies on inertial sensors to estimate a user’s trajectory, has gained prominence due to its independence from external infrastructure. However, PDR suffers from cumulative errors over time, necessitating the integration of complementary techniques to maintain accuracy [8,9,10].

Another widely utilized approach is signal fingerprinting, which maps signal characteristics to specific indoor locations. While effective in controlled environments, its performance significantly deteriorates in dynamic or obstructed settings [11,12,13]. Recent studies have combined PDR with signal fingerprinting to harness their respective strengths. For example, Cheng et al. [14] introduced an implicit unscented particle filter to fuse sensor data, achieving moderate accuracy improvements. However, these methods often face scalability, computational efficiency, and adaptability constraints, particularly in real-time applications [9,14,15,16].

This study proposes a novel multi-source fusion algorithm that combines PDR and signal fingerprinting using a weighted fusion framework. Unlike previous methods, this one uses new techniques for separating different types of pedestrian motion states and flexible strategies that let it adapt to changing conditions on the fly. This makes it much more robust and efficient at using computers. By addressing the limitations of single-source systems and enhancing accuracy, the proposed approach demonstrates superior performance over existing methods, including standalone PDR, signal fingerprinting, and previously combined techniques [12,17]. Experimental evaluations highlight its scalability and applicability in complex and dynamic indoor environments, underscoring its practical significance. We organize the remainder of this paper as follows: Section 2 reviews related work on multi-source fusion techniques and indoor positioning challenges. Section 3 describes the methodology of the proposed algorithm, including its motion state segmentation and fusion framework. Section 4 presents experimental results and comparative analyses, while Section 5 discusses implications, limitations, and future directions. The conclusions are presented in Section 6.

## 2. Related Work

Due to their simplicity and availability, single-source positioning systems, such as Wi-Fi, Bluetooth, and ultra-wideband (UWB), are widely employed for indoor localization. Wi-Fi fingerprinting relies on received signal strength indicators (RSSIs) to estimate positions but suffers from environmental interference and signal variability [13,18]. Similarly, Bluetooth-based systems offer high spatial resolution but are vulnerable to signal attenuation in crowded environments [14,15]. UWB systems, while providing high-precision positioning, face challenges in scalability and cost-effectiveness for large-scale deployments [19,20,21].

Inertial sensors, particularly those integrated into wearable devices, have emerged as robust tools for pedestrian positioning via pedestrian dead reckoning (PDR). PDR estimates step length, frequency, and heading direction to provide continuous localization independent of external signals [22]. However, PDR is inherently affected by cumulative errors stemming from sensor drift [10,23]. Recent advancements have employed advanced filtering techniques, such as particle and Kalman filters, to mitigate these errors and improve the accuracy of PDR-based systems [14,20].

Signal fingerprinting, which maps signal features such as RSSIs to specific indoor locations, is renowned for its accuracy in controlled environments [24]. Nevertheless, its reliance on static signal characteristics renders it less effective in dynamic and obstructed scenarios [5,25]. Recent research has explored integrating additional signal modalities, such as geomagnetic fields and Bluetooth low energy (BLE), to enhance localization accuracy [11,17].

Multi-source fusion has gained prominence as a strategy to overcome the limitations of single-source systems. By combining data from inertial sensors and signal fingerprints, researchers have developed methods that leverage the complementary strengths of these modalities. For instance, Wang et al. [5] proposed a hybrid approach that integrates PDR with Wi-Fi fingerprinting through an improved particle filter, achieving significant performance gains. Similarly, Hua et al. [11] introduced a deep learning-based BLE-IMU fusion system, demonstrating strong adaptability to dynamic environments despite high computational requirements [26]. Fusion methods utilizing Kalman filters, unscented particle filters, and neural networks have been widely adopted to enhance the robustness and precision [27,28,29]. However, they require careful parameter tuning to adapt to varying environmental conditions [20,30].

Hu and Assaad et al. [25] proposed a BIM-enabled digital twin framework, integrating LiDAR-based 3D mapping, IoT sensing, and autonomous robotics for real-time indoor positioning and environment monitoring. Their approach enhances spatial awareness and adaptive localization, making it valuable for smart buildings and industrial automation. However, challenges such as high computational overhead and sensor calibration remain. This integration demonstrates the potential of digital twins in dynamic indoor localization, enabling real-time decision-making and predictive analytics.

Senel et al. [31] proposed a real-time multi-object tracking framework that integrates data from multiple sensors, including LiDAR, radar, and cameras. Their approach employs an unscented Kalman filter (UKF) alongside an object association module based on the Hungarian algorithm, ensuring robust tracking performance across varying environmental conditions. This study highlights the significance of multi-sensor fusion in improving tracking accuracy and reliability, particularly in dynamic and occluded indoor environments. However, challenges remain regarding real-time computational efficiency and sensor deployment scalability, which are crucial considerations for large-scale positioning applications.

Another approach that enhances localization performance involves fusing data from multiple inertial sensors using machine learning techniques; Song et al. [3] proposed a positioning method that integrates data from multiple wearable inertial sensors. Their approach enhances localization accuracy by dynamically adapting to variations in user motion and environmental conditions. While this method improves robustness compared to single-node PDR, it introduces computational complexity and scalability challenges, particularly in large-scale indoor environments.

Wang and Zhang [32] proposed a robust sensor fusion framework for indoor wireless localization to address the challenges posed by multi-path fading and non-line-of-sight (NLoS) conditions. Their method integrates Zigbee wireless sensor networks (WSNs) with received signal strength (RSS) measurements, leveraging an extended Kalman filter (EKF) to improve localization accuracy. The system uses multiple mobile nodes equipped with inertial sensors (ISs) to estimate their roll, pitch, and yaw angles, enabling them to model movement paths as curves. Their method effectively reduces localization distortion and enhances robustness in heavily obstructed environments by utilizing cooperative positioning between mobile nodes.

However, the reliance on predefined node configurations limits adaptability in dynamic environments, and the computational cost of EKF-based fusion makes it less suitable for real-time applications in large-scale deployments. Compared to this approach, the proposed work in this study incorporates adaptive pedestrian motion segmentation and dynamic fusion strategies to balance computational efficiency and accuracy, making it more scalable for real-world deployment.

Despite significant advancements, existing indoor positioning systems (IPSs) face several critical limitations that hinder their performance in dynamic and real-world scenarios. These limitations include environmental variability, computational efficiency, scalability, and adaptability challenges.

Recent works, such as by Cheng et al. [14], introduced an implicit unscented particle filter (I-UPF) to fuse inertial and signal data, achieving moderate accuracy improvements. However, the computational cost of particle filter-based methods often limits their applicability in real-time systems. Wang et al. [5] utilized deep learning to predict user positions from multi-modal data sources, including Wi-Fi and inertial measurements. While promising, these methods require extensive training data and computational resources, making them less practical for dynamic, large-scale deployments. Furthermore, works such as by Milano et al. [15] and Tang et al. [13] have explored BLE and Wi-Fi RSSI-based augmentation techniques to improve localization accuracy, demonstrating the increasing role of signal fusion in multi-modal indoor positioning. Additionally, Álvarez-Merino et al. [7] compared Wi-Fi, 5G, and hybrid fusion localization under incomplete map conditions, showing that integrating multiple wireless technologies significantly improves positioning robustness and adaptability. A summary of these approaches, along with their techniques and limitations, is provided in Table 1.

Computational complexity and real-time feasibility: Many high-accuracy positioning methods rely on particle filters, deep learning, and multi-sensor fusion, which introduce high computational overhead. Approaches such as strong tracking particle filters [2] and unscented particle filters [14] achieve accuracy improvements but remain unsuitable for real-time applications due to their intensive computational requirements. Similarly, deep learning-based fusion techniques [11] demand large training datasets and computational power, limiting their scalability for dynamic, large-scale environments.

Sensitivity to environmental variations: Several IPS techniques, including RSSI-based Wi-Fi fingerprinting and BLE localization, are highly sensitive to signal fluctuations, multipath interference, and obstructions. Studies such as by Tang et al. [13] and Milano et al. [15] highlight the instability of RSSI and BLE signals in real-world environments. Additionally, LiDAR and infrared-based approaches [33] suffer from lighting condition dependencies, further complicating deployment.

Scalability and infrastructure constraints: High-precision methods such as UWB, LiDAR-based mapping, and sensor fusion require extensive infrastructure investments, making them difficult to scale in large, complex environments. Works such as by Sun et al. [20] and Hu and Assaad et al. [25] discuss how 3D mapping and IoT-based digital twins significantly increase data processing requirements, making real-time positioning computationally intensive.

Sensor misalignment and fusion challenges: Multi-sensor fusion techniques have improved IPS accuracy, but they introduce alignment and synchronization challenges. Methods such as robust sensor fusion [32] and multi-object tracking using real-time sensor fusion [31] experience accuracy degradation due to sensor drift and misalignment. This issue becomes critical in scenarios where precise trajectory tracking is required.

Incomplete mapping and localization uncertainty: Localization techniques that rely on map-based positioning suffer from incomplete or outdated mapping data, leading to accuracy degradation. Alvarez-Merino et al. [7] demonstrate how 5G-based localization, despite its advantages, is significantly affected by incomplete maps. Similarly, PIR and ultrasonic-based people-tracking approaches [17] experience limitations when line-of-sight conditions are unmet.

Proposed solution: This study introduces a fusion algorithm that dynamically integrates PDR and wireless signal fingerprinting to address these shortcomings. The proposed approach mitigates cumulative errors, adapts to dynamic environments, and maintains computational efficiency by incorporating motion state recognition and an adaptive weighting framework. Experimental evaluations demonstrate the algorithm’s robustness under diverse conditions, highlighting its scalability and practical applicability.

## 3. Materials and Methods

### 3.1. Pedestrian Dead Reckoning Based on a Six-Axis Inertial Sensor

Pedestrian dead reckoning (PDR) is a widely used technique in indoor positioning systems due to its ability to provide continuous trajectory estimation without relying on external signals [34,35]. By leveraging data from six-axis inertial sensors, including accelerometers and gyroscopes, PDR calculates step length, heading direction, and step frequency to estimate a pedestrian’s position [36,37,38]. This approach is particularly effective when wireless signal availability is limited or unreliable.

The proposed method, the fusion positioning algorithm based on inertial sensors and signal fingerprints, integrates two key components: pedestrian dead reckoning (PDR) based on a six-axis inertial sensor and a fusion positioning algorithm based on pedestrian state recognition. This section presents the preliminaries and methodology.

**Figure 1:**
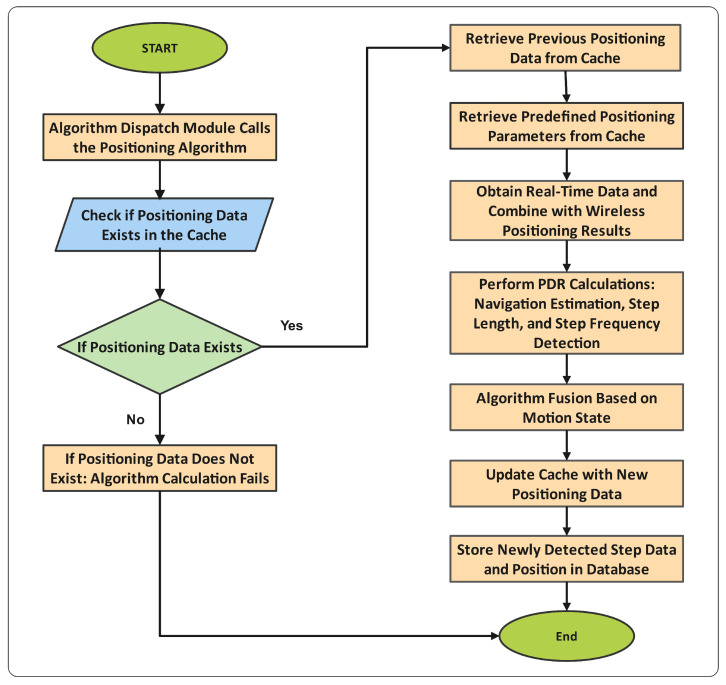
Algorithm execution module. The flowchart illustrates the step-by-step process of the proposed algorithm, including data retrieval, preprocessing, PDR calculations, motion state-based fusion, and final localization updates.

Figure 1 illustrates the detailed workflow of the proposed methodology. The process begins by retrieving prior positioning data from the cache’s predefined positioning parameters, including location, timestamp, and heading angle. Real-time sensor data are combined with wireless signal positioning results to perform key PDR calculations such as trajectory estimation, step frequency detection, and step length estimation. Based on motion state recognition, the algorithm dynamically applies a fusion framework that uses weighted integration to adjust for linear motion or turns. Updated positioning data are stored back in the cache, and newly detected step data are added to the database, ensuring adaptability for subsequent calculations. If required data are missing in the cache, the algorithm terminates the process to prevent errors. This systematic workflow ensures robust and accurate indoor localization, even in dynamic environments.

#### 3.1.1. Pedestrian Dead Reckoning (PDR)

Pedestrian dead reckoning (PDR) is a widely used positioning method based on inertial sensors. This approach determines a pedestrian’s step frequency, length, and heading direction in real time by analyzing inertial sensor readings from a wearable device carried by the pedestrian [34]. PDR predicts the pedestrian’s next position using the known current position, enabling continuous updates to their location.

**Figure 2:**
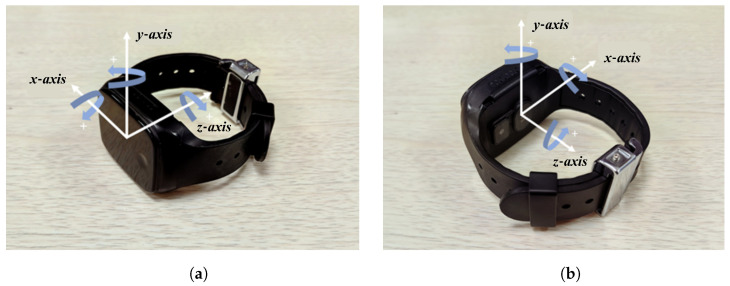
Wristband coordinate-system illustration showing the axes configuration visible from the front and back. (**a**) Front view of the wristband coordinate system. (**b**) Back view of the wristband coordinate system.

Figure 2 illustrates the coordinate system of the positioning wristband from both back and front perspectives. The direction of the arrows represents the positive direction of acceleration or angular velocity. During actual pedestrian movement, the positioning wristband is worn on the pedestrian’s wrist and swings with the arm. This means that the inertial sensor’s coordinate system does not coincide with the pedestrian’s coordinate system, and their relative positions change continuously. As a result, the wristband’s motion state does not directly reflect the pedestrian’s motion state. Therefore, a pedestrian dead reckoning (PDR) algorithm that adapts to the current arm-swing state is required.

The PDR algorithm models the pedestrian’s trajectory as a series of line segments, each representing a single step. Let the position of the pedestrian at time t−1 be $\left(x_{t-1},y_{t-1}\right)$, the step length during the interval from t−1 to *t* be $l_{t-1}$, and the heading direction angle at t−1 be $\theta_{t-1}$. The estimated position of the pedestrian at time *t*, denoted as $\left(x_{t},y_{t}\right)$, can be calculated using the following formulas:

(1) $$x_{t}=x_{t-1}+l_{t-1}\cdot \cos\left(\theta_{t-1}\right),$$

(2) $$y_{t}=y_{t-1}+l_{t-1}\cdot \sin\left(\theta_{t-1}\right),$$

The pedestrian’s movement can be modeled by establishing a relationship between the inertial sensor’s acceleration and angular velocity readings and the pedestrian’s step frequency, step length, and heading direction. Using this model, the PDR algorithm can estimate the pedestrian’s position.

To compute the next position, it is necessary to determine the time *t* of the next step, the step length $l_{t-1}$, and the heading direction angle $\theta_{t-1}$. These three values correspond to the core tasks of the PDR algorithm:

1. Step frequency detection: Identifying the frequency of periodic peaks in the acceleration waveform.
2. Step length estimation: Estimating the step length based on a linear or empirical model.
3. Heading estimation: Calculating the heading direction angle using angular velocity data and quaternion-based orientation updates.

The inertial sensor used in this study is embedded in a positioning wristband. It is a six-axis inertial sensor capable of measuring accelerations along the *x*, *y*, and *z* axes, as well as angular velocities around these axes. The wristband’s coordinate system is depicted in Figure 2.

#### 3.1.2. Step Frequency Detection

Step frequency detection involves identifying a pedestrian’s step frequency using sensor data. Fundamentally, it establishes a relationship between the periodic waveform of sensor readings and the pedestrian’s walking cycle to estimate step frequency.

In most PDR research literature, the inertial sensor is fixed relative to the pedestrian, and its coordinate axes are aligned with the pedestrian’s coordinate system [31,33]. Based on walking mechanics, the *z*-axis acceleration typically exhibits periodic peaks corresponding to the pedestrian’s steps. Therefore, detecting the peaks in the *z*-axis acceleration effectively determines step frequency.

In this study, the inertial sensor’s coordinate axes are not aligned with the pedestrian’s coordinate system. Instead, the sensor swings continuously while walking with the pedestrian’s arm, making the situation more complex. During arm swinging, the arm’s position relative to the body continuously changes, making it challenging to use acceleration in a single direction to represent the pedestrian’s motion cycle. Therefore, we consider observing the waveform of the resultant acceleration as it changes over time. This section discusses step frequency detection under conditions where the inertial sensor moves with the arm swing.

**Figure 3:**
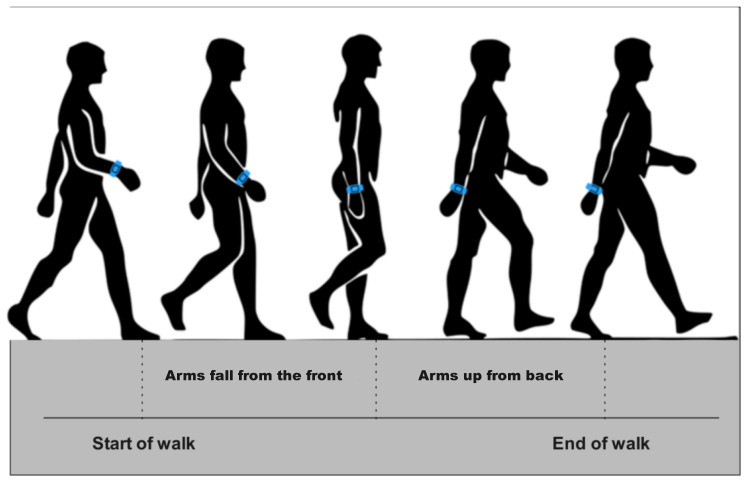
Relationship between pedestrian steps and arm motion. The arm swing follows a periodic pattern as it moves from the front to the back during walking, synchronized with the pedestrian’s steps.

The relationship between the pedestrian’s gait and arm swing is shown in Figure 3. During a single step, the right hand of the pedestrian moves from the front drop to a position perpendicular to the ground and then swings backward.

**Figure 4:**
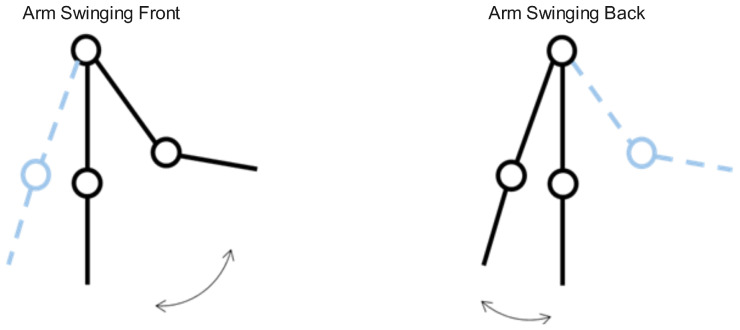
Arm swinging model: (**left**) Arm swinging forward with larger amplitude; (**right**) arm swinging backward with smaller amplitude. The periodic motion is synchronized with the pedestrian’s gait.

The arm swing during walking can be abstracted into the model shown in Figure 4. Due to the anatomical structure of the arm’s joints, the swing amplitude is larger in front of the body and smaller behind the body. Furthermore, the arm movement is synchronized with the steps, and the time spent swinging forward and backward is theoretically the same. As a result, the arm moves faster when swinging forward and slower when swinging backward. Additionally, as the forearm drops, the wristband experiences a noticeable weightlessness effect during the transition from swinging forward to backward.

The resultant acceleration across three axes is calculated as follows:

(3) $$a=\sqrt{a_{x}^{2}+a_{y}^{2}+a_{z}^{2}},$$

where *a* is the resultant acceleration and $a_{x}$, $a_{y}$, and $a_{z}$ are the accelerations along the *x*, *y*, and *z* axes.

The waveform of the resultant acceleration over a 6-meter straight trajectory was observed to identify its relationship with the pedestrian’s walking cycle. The wristband was worn on the left wrist, and the arm swung naturally during normal walking. The initial position was defined with both feet together, and the first step was taken with the right foot. A “step” is where one foot remains on the ground while the other moves from lifting off to touching down again. According to this definition, step length detection focuses on the same foot. For this trajectory, there were five steps with the left foot and six with the right foot, with each step manually set to a fixed length of 120 cm.

**Figure 5:**
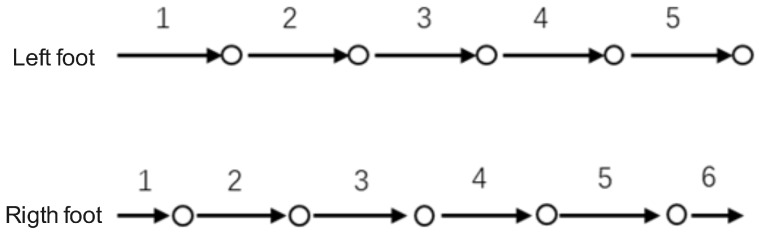
Schematic diagram of step points over a 6 m straight trajectory. The diagram illustrates the positions of each footfall during normal walking.

A schematic diagram of the step points is shown in Figure 5.

**Figure 6:**
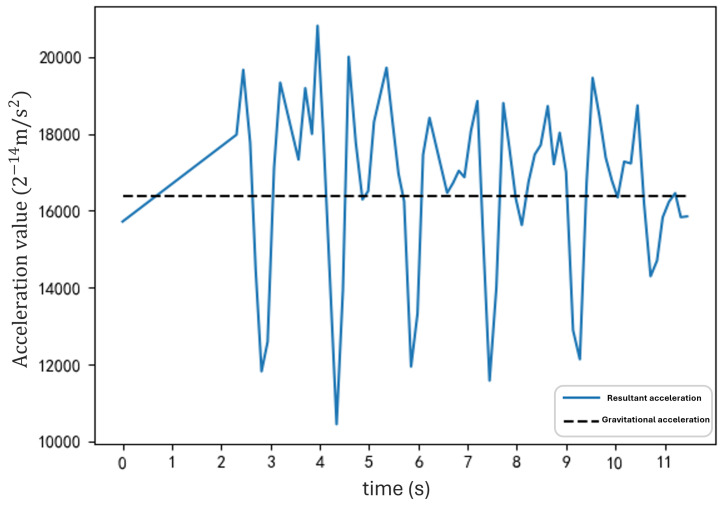
Resultant acceleration waveform during walking. The blue line represents the resultant acceleration readings, and the black dashed line indicates the gravitational acceleration *g* (9.8 m/s^2^). Each “M”-shaped fluctuation corresponds to a step, with prominent troughs observed when the arm swings downward and experiences a weightlessness effect.

The resultant acceleration waveform obtained by walking along the defined step points is shown in Figure 6.

Figure 6 shows that the blue line represents the resultant acceleration readings, and the black dashed line indicates the gravitational acceleration *g*, taken here as 9.8 m/s^2^. The waveform exhibits apparent periodicity over time, with each step corresponding to an “M”-shaped fluctuation. Additionally, there are prominent troughs on either side of each “M”-shaped fluctuation. During these troughs, the resultant acceleration readings from the inertial sensor fall below the gravitational acceleration [39]. This occurs because, as the arm swings downward from the front of the body, the inertial sensor experiences a weightlessness effect, reducing the acceleration readings and lowering the calculated resultant acceleration [40,41].

**Figure 7:**
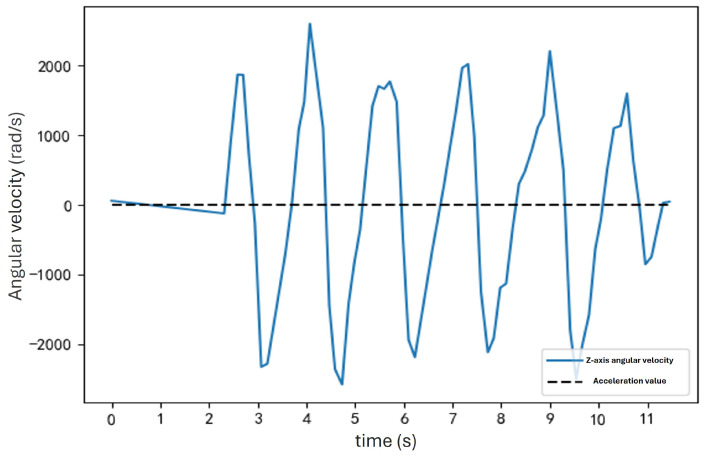
Waveform of the *z*-axis angular velocity during walking. The *z*-axis angular velocity aligns with the arm swing’s direction, effectively representing the pedestrian’s arm swing angular velocity and showing strong periodicity.

Next, observe the waveform of the *z*-axis angular velocity during pedestrian walking, as shown in Figure 7.

During arm swinging, the inertial sensor’s *z*-axis angular velocity aligns with the arm swing’s direction. Thus, the *z*-axis angular velocity effectively represents the angular velocity of the pedestrian’s arm swing and exhibits strong periodicity.

**Figure 8:**
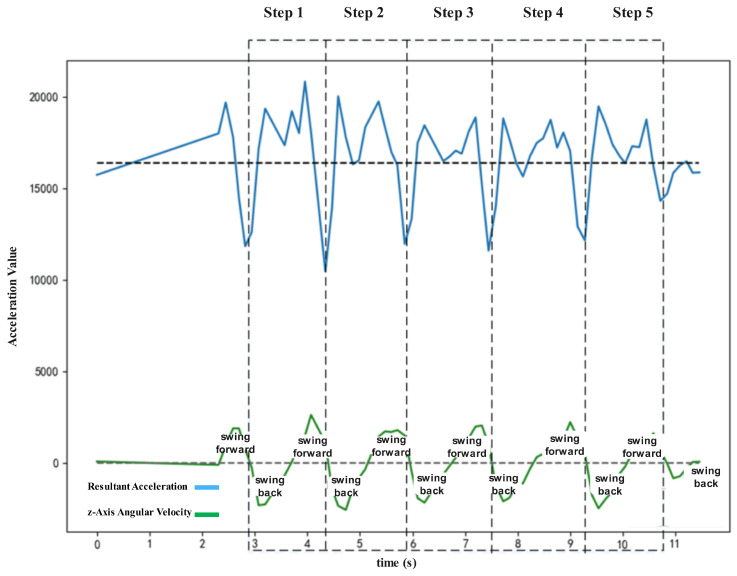
Comparison of resultant acceleration and *z*-axis angular velocity waveforms. Each step corresponds to a complete cycle of both waveforms. The vertical dashed lines mark the five steps taken during the observed walking trajectory.

By comparing the waveforms of resultant acceleration and *z*-axis angular velocity, the relationship between pedestrian step frequency and sensor waveforms can be analyzed. Both waveforms are plotted on the same graph, as shown in Figure 8. In the figure, the two horizontal dashed lines represent the gravitational acceleration value and the zero value of angular velocity, serving as reference lines to help observe waveform changes. The blue curve represents the resultant acceleration values, while the green curve represents the *z*-axis angular velocity values.

The comparison in Figure 8 shows the changes in the resultant acceleration waveform and *z*-axis angular velocity during a left-foot step. Specifically:

- When the left hand transitions from swinging forward to swinging backward, the *z*-axis angular velocity crosses zero, and the resultant acceleration reaches a trough. At this moment, the left foot takes a step forward.
- During the transition of the left hand from swinging backward to forward, the left foot remains stationary, supporting the body, while the right foot moves forward [14].
- When the left hand swings forward and then transitions to swinging backward again, the *z*-axis angular velocity crosses zero, and the resultant acceleration reaches another trough. The left foot then takes another step forward.

Figure 8 demonstrates that the pedestrian’s five steps correspond precisely to the five cycles of the inertial sensor’s resultant acceleration and *z*-axis angular velocity. At this point, the pedestrian’s walking state can be linked to the inertial sensor readings. Step frequency detection can be completed by identifying the sensor’s acceleration or angular velocity waveforms.

This chapter employs a sliding window method to identify the troughs in the resultant acceleration waveform. The time intervals between successive troughs are then used to calculate the pedestrian’s walking frequency. Specifically, suppose the resultant acceleration value at the center of the sliding window is smaller than the values of all other samples within the window. In that case, this point is identified as a trough.

#### 3.1.3. Step Length Estimation

Step length estimation uses sensor data to estimate the pedestrian’s next step length. Standard step length estimation models include constant, linear, and nonlinear models. Considering these models’ applicability and computational complexity, this study adopts a linear model to estimate the pedestrian’s step length.

The linear model assumes that the step length is linearly related to the step frequency. The faster the pedestrian’s step frequency, the longer the step length. The calculation formulas are as follows:

(4) $${\mathrm{StrideLength}}_{k}=\alpha \cdot {\mathrm{freq}}_{k}+\beta$$

(5) $${\mathrm{freq}}_{k}=\frac{1}{t_{k}-t_{k-1}}$$

where ${\mathrm{freq}}_{k}$ is the step frequency of the *k*-th step, $t_{k}$ is the time the *k*-th step occurs, and α and β are the two parameters of the linear model, both constants. The parameter α represents the proportionality constant linking step frequency and step length, while β accounts for a baseline step length when the step frequency is minimal or zero.

#### 3.1.4. Heading Estimation

Heading estimation involves calculating the pedestrian’s current heading direction based on sensor data. It is the most critical and complex problem in the PDR algorithm.

In this study, the inertial sensor is worn on the pedestrian’s wrist and moves with the swinging arm. Consequently, its motion direction differs from the forward direction of the pedestrian’s torso, adding complexity to heading estimation. Under such motion conditions, academic research frequently employs quaternion-based methods to represent the wristband’s orientation angles. The quaternion matrix is updated using angular velocity data, simplifying the calculations for orientation angles (pitch, roll, yaw) around the *x*, *y*, and *z* axes. The quaternion vector ${\left[q_{1},q_{2},q_{3},q_{4}\right]}^{T}$ represents the inertial sensor’s orientation angles. This updated quaternion matrix is then used to calculate the sensor’s orientation angles.

However, since the sensor is worn on the pedestrian’s wrist, additional calculations are required to derive the pedestrian’s heading direction from the sensor’s orientation angles. Observations indicate that during standard arm swinging while walking, the *x*-axis of the wristband’s coordinate system, when projected onto the horizontal plane (xOy) of the reference coordinate system, always points opposite the pedestrian’s forward direction. Thus, the pedestrian’s heading direction can be calculated by determining the direction of the wristband’s *x*-axis in the reference coordinate system.

To determine the transformation from the wristband coordinate system to the reference coordinate system, the rotation matrix $R_{b}^{n}$ is calculated as follows:

(6) $$R_{b}^{n}=\left[\begin{array}{ccc} \cos\mathrm{Yaw} & -\sin\mathrm{Yaw} & 0\\ \sin\mathrm{Yaw} & \cos\mathrm{Yaw} & 0\\ 0 & 0 & 1 \end{array}\right]\left[\begin{array}{ccc} 1 & 0 & 0\\ 0 & \cos\mathrm{Pitch} & -\sin\mathrm{Pitch}\\ 0 & \sin\mathrm{Pitch} & \cos\mathrm{Pitch} \end{array}\right]\left[\begin{array}{ccc} \cos\mathrm{Roll} & 0 & \sin\mathrm{Roll}\\ 0 & 1 & 0\\ -\sin\mathrm{Roll} & 0 & \cos\mathrm{Roll} \end{array}\right]$$

Assume there is a vector $v_{\mathrm{tag}}$ in the wristband coordinate system aligned with the *x*-axis. Its representation in the reference coordinate system is expressed as follows:

(7) $$v_{\mathrm{ref}}=R_{b}^{n}\cdot v_{\mathrm{tag}}$$

where $R_{b}^{n}$ is the rotation matrix, transforming the wristband coordinate system to the reference coordinate system, and $v_{\mathrm{ref}}$ represents the coordinates of the vector in the reference system. By projecting $v_{\mathrm{ref}}$ onto the xOy plane, the pedestrian’s heading angle can be obtained.

The angular displacement projection method simplifies heading estimation in indoor pedestrian scenarios. This method uses the wristband’s angular displacements to project onto the pedestrian’s heading direction. The angular displacements are computed by integrating the angular velocities along the three axes:

(8) $$\theta_{x}=\int_{t_{\mathrm{begin}}}^{t_{\mathrm{stop}}}\omega_{x}\;dt,\;\theta_{y}=\int_{t_{\mathrm{begin}}}^{t_{\mathrm{stop}}}\omega_{y}\;dt,\;\theta_{z}=\int_{t_{\mathrm{begin}}}^{t_{\mathrm{stop}}}\omega_{z}\;dt,$$

where $\theta_{x}$, $\theta_{y}$, and $\theta_{z}$ represent the angular displacements around the *x*, *y*, and *z* axes, respectively, and $\omega_{x}$, $\omega_{y}$, and $\omega_{z}$ are the angular velocity components along these axes. $t_{\mathrm{begin}}$ and $t_{\mathrm{stop}}$ denote the start and end times of the integration period.

By projecting the angular displacements onto the pedestrian’s heading plane, the change in heading angle is calculated as follows:

(9) $$\theta_{Z}=\frac{a_{x}\theta_{x}+a_{y}\theta_{y}+a_{z}\theta_{z}}{\sqrt{a_{x}^{2}+a_{y}^{2}+a_{z}^{2}}},$$

where $\theta_{Z}$ represents the heading angle, and $a_{x}$, $a_{y}$, and $a_{z}$ are the acceleration components along the *x*, *y*, and *z* axes.

This method is more computationally efficient than quaternion-based updates. In this scenario, only the pedestrian’s forward direction is required, making the angular displacement projection method more practical for indoor navigation. PDR provides continuous trajectory tracking and does not rely on external infrastructure, making it suitable for diverse indoor environments. However, its accuracy is limited by cumulative errors due to sensor drift over time. This necessitates integrating complementary methods, such as signal fingerprinting, to mitigate these errors.

### 3.2. Fusion Positioning Algorithm Based on Pedestrian State Recognition

#### 3.2.1. Pedestrian Motion State Machine

The PDR algorithm has the advantage of accurately tracking a pedestrian’s trajectory; however, it requires a predefined starting point and suffers from cumulative errors. Wireless signal fingerprinting can provide relatively accurate positional information but cannot precisely track the pedestrian’s trajectory. Additionally, it may experience significant errors at specific points due to indoor obstacles.

**Figure 9:**
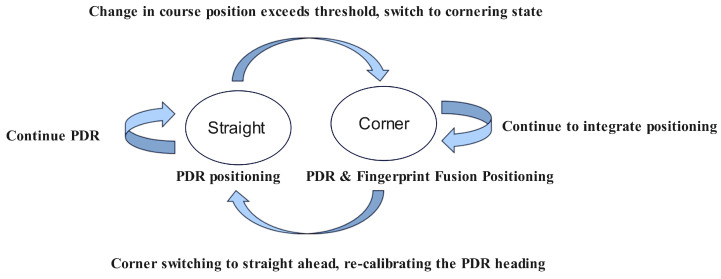
Pedestrian motion state machine. This diagram illustrates the proposed methodology’s dynamic switching between straight-line motion and cornering states. During straight-line motion, PDR is used for trajectory tracking. When the change in heading angle exceeds a predefined threshold, the system transitions to a cornering state where PDR is fused with wireless signal fingerprinting for enhanced positioning accuracy. Upon resuming straight-line motion, the PDR heading is recalibrated.

In the algorithm proposed in this research, the pedestrian’s indoor movement is abstracted into linear motion and turning. The pedestrian’s motion along straight trajectories and during turns is discussed separately [42]. First, pedestrian states are determined based on heading and step frequency information. Then, different fusion positioning methods are applied according to the pedestrian’s state to leverage the strengths of both positioning approaches. The pedestrian motion state machine is shown in Figure 9.

Since step frequency detection can distinguish between walking and stationary states, the stationary state is not included in the pedestrian motion state machine for discussion. When the pedestrian’s heading fluctuations are smaller than a certain threshold, they are considered normal signal variations, and the pedestrian is classified as being in a linear motion state. In this case, no adjustments are made to the heading in the PDR algorithm. During linear motion, the PDR algorithm continues to be used for positioning.

When the pedestrian’s heading changes exceed the threshold, the algorithm classifies the pedestrian as making a turn, requiring a heading adjustment. Because the PDR algorithm’s heading estimation relies entirely on sensor readings, it may struggle to accurately capture the pedestrian’s turning angle during sharp heading changes, potentially resulting in positioning errors.

To address this, the algorithm fuses the positioning results from the PDR algorithm and wireless signal fingerprinting at turning points to calculate the user’s position. The fusion method uses a weighted average of the two positioning results, with the calculation formula as follows:

(10) $${\mathrm{pos}}_{\mathrm{mix}}={\mathrm{pos}}_{\mathrm{signal}}\cdot w+{\mathrm{pos}}_{\mathrm{pdr}}\cdot \left(1-w\right),$$

where ${\mathrm{pos}}_{\mathrm{mix}}$ represents the fused positioning coordinate, which is a two-dimensional vector containing the *x* and *y* coordinates. ${\mathrm{pos}}_{\mathrm{signal}}$ and ${\mathrm{pos}}_{\mathrm{pdr}}$ are the coordinates obtained from wireless signal positioning and PDR algorithm calculations, respectively. ω is the weight for the weighted average, with a value ranging between 0 and 1 [40,41].

Derivation of *possignal*: The term ${\mathrm{pos}}_{\mathrm{signal}}$ is derived from wireless signal fingerprinting. Signal fingerprints, such as received signal strength indicators (RSSIs), are collected from multiple access points and matched against a pre-constructed fingerprint database. The closest match in the database determines the estimated position, ${\mathrm{pos}}_{\mathrm{signal}}$, which is then integrated with the PDR estimate through the weighted fusion framework.

Calculation of *w*: The weight *w* in Formula (10) is calculated to minimize the Mean Squared Error (MSE) between the fused position (${\mathrm{pos}}_{\mathrm{mix}}$) and the ground truth position (${\mathrm{pos}}_{\mathrm{true}}$). The formula for determining *w* is as follows:

(11) $$w=\frac{\sum_{i=1}^{n}\left({\mathrm{pos}}_{\mathrm{signal},i}-{\mathrm{pos}}_{\mathrm{pdr},i}\right)\cdot \left({\mathrm{pos}}_{\mathrm{true},i}-{\mathrm{pos}}_{\mathrm{pdr},i}\right)}{\sum_{i=1}^{n}{\left({\mathrm{pos}}_{\mathrm{signal},i}-{\mathrm{pos}}_{\mathrm{pdr},i}\right)}^{2}},$$

where

- *n*: Number of data points in the trajectory.
- ${\mathrm{pos}}_{\mathrm{signal},i}$: Position estimated by wireless signal fingerprinting for the *i*-th step.
- ${\mathrm{pos}}_{\mathrm{pdr},i}$: Position estimated by PDR for the *i*-th step.
- ${\mathrm{pos}}_{\mathrm{true},i}$: Ground truth position for the *i*-th step.

For the experiments conducted in this study, the value of *w* was approximately 0.67 for tested trajectories, balancing the contributions of PDR and signal fingerprinting.

Significance of the Fusion Method: The fusion approach compensates for the cumulative drift in PDR by incorporating signal fingerprinting, particularly during turns. The adaptive weighting mechanism ensures that PDR dominates during straight-line motion for continuous tracking, while signal fingerprinting corrects positioning errors during complex turns. This dynamic adjustment leverages the strengths of both positioning techniques, achieving enhanced accuracy and robustness in dynamic indoor environments [29,39].

If the pedestrian’s heading change falls below the predefined threshold in the next time window, the algorithm classifies the pedestrian as having returned to a linear motion state. The heading is re-calibrated, and the positioning method switches back to relying solely on the PDR algorithm.

#### 3.2.2. Turn Detection

A time-domain rolling window mechanism is designed to identify pedestrian turns. Assume that each rolling window contains *m* consecutive samples over a defined time interval, with each sample including three-axis acceleration and angular velocity data for a specific moment. Different rolling windows do not share overlapping samples. The information from a single rolling window is sufficient for identifying pedestrian turns.

If the time span within the rolling window is too short, it may result in false turn detection; if it is too long, turn detection may be delayed. Typically, a time span of 1–2 s is suitable. For example, in the system deployed for this algorithm, the data push frequency is 5 Hz, meaning the time interval between adjacent samples is 0.2 s. Thus, the rolling window size is set to 7 samples, resulting in a time span of 1.4 s.

**Figure 10:**
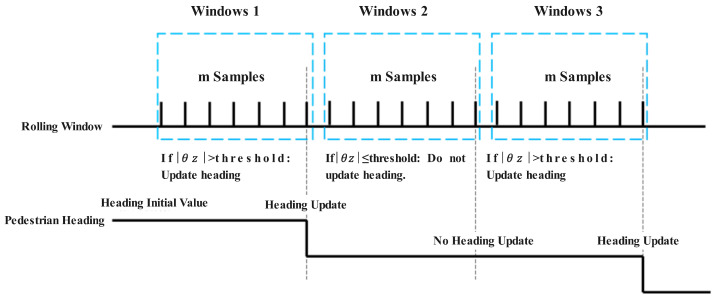
Schematic diagram of turn detection using a rolling window. The figure illustrates the rolling window mechanism and the conditions for updating the heading during detected turns.

As shown in Figure 10, if the absolute value of the cumulative angular displacement $|\theta_{Z}|$ within a rolling window exceeds the predefined threshold, this angular displacement is used to update the heading. Otherwise, the heading remains unchanged. This approach aims to avoid errors in heading estimation caused by angular velocity fluctuations during linear motion. It ensures that heading updates only occur during turns, resulting in more precise positioning.

During linear motion, the algorithm assumes that the PDR method can accurately track the pedestrian’s position. Therefore, it directly uses the PDR algorithm for positioning without referring to the results of a wireless signal. However, when the turn recognition algorithm identifies a turning state, it assumes that the PDR method performs poorly at turns. To address this, the algorithm combines PDR results with wireless signal positioning using a weighted fusion method to determine the final position.

#### 3.2.3. Heading Reset

**Figure 11:**
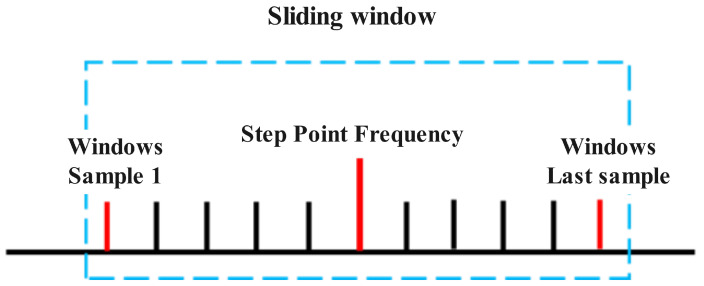
Schematic diagram of heading reset using a sliding window. This figure illustrates the recalibrating of the pedestrian’s heading during the transition back to linear motion.

When a pedestrian transitions from a turning state to a linear motion state, the algorithm switches back to PDR-based positioning. At this point, resetting the pedestrian’s heading is necessary to obtain the initial direction for the subsequent linear motion. As shown in Figure 11, the heading reset process utilizes a sliding window approach to recalibrate the pedestrian’s direction during this transition. This ensures smoother trajectory estimation and improved positioning accuracy.

The heading reset is achieved using a sliding window mechanism, which is used for step frequency detection. The current step’s central point lies in the middle of the sliding window, with several samples on either side. The new heading is calculated based on the coordinates of the first and last samples in the sliding window. The formula is as follows:

(12) $$\theta =\arctan\left(\frac{y_{\mathrm{last}}-y_{\mathrm{first}}}{x_{\mathrm{last}}-x_{\mathrm{first}}}\right),$$

where $\left(x_{\mathrm{first}},y_{\mathrm{first}}\right)$ represents the coordinates of the first sample in the sliding window, and $\left(x_{\mathrm{last}},y_{\mathrm{last}}\right)$ represents the coordinates of the last sample in the sliding window [43,44].

The heading can be recalibrated using the formula provided, allowing the system to switch back to PDR-based positioning in the linear motion state. This ensures accurate positioning for subsequent trajectories in the pedestrian’s movement [23,41].

## 4. Experiment Results

### 4.1. Experiment Setup

**Figure 12:**
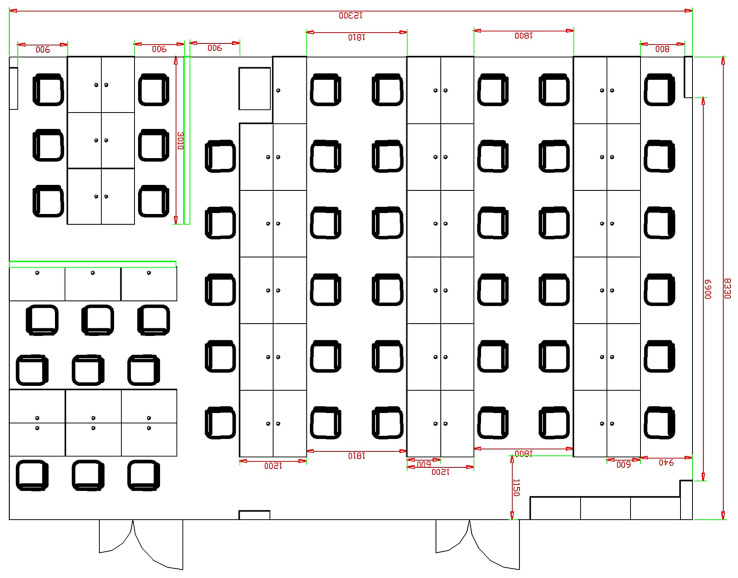
Schematic of the experimental environment. The laboratory space includes common office furniture and freely moving individuals, simulating real-world conditions.

The experiment and data used in this study were conducted and collected in a laboratory environment at a university building. The laboratory, measuring 12.3 m × 8.6 m, represents a typical indoor office space containing desks, chairs, and computers. During the experiment, people moved freely within the area, simulating real-world indoor positioning scenarios. A schematic of the experimental environment is shown in Figure 12.

Wireless signal fingerprinting in the experiment was conducted using Wi-Fi signals, leveraging the received signal strength indicators (RSSIs) collected from multiple access points distributed throughout the laboratory space. Wi-Fi was selected as the signal source due to its widespread availability in indoor environments and compatibility with the fusion-based localization framework. The RSSI data were integrated with inertial sensor measurements to create a signal map for localization experiments.

**Figure 13:**
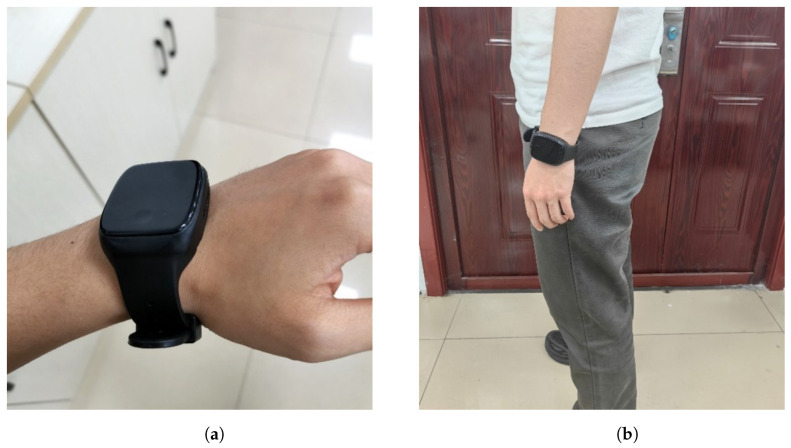
Photographs illustrating the wristband-wearing configuration during the experiment. The left image (**a**) shows the full setup, while the right image (**b**) provides a close-up of the wearable device.

The signal acquisition device used in the experiment was a wearable wristband equipped with a six-axis inertial sensor. The wristband was worn on the left wrist of the participant and moved along with the natural arm swing during walking, as shown in Figure 13.

Four sampling paths, three U-shaped and one Z-shaped, were designed to verify the algorithm’s effectiveness. Five forward trajectories and five reverse trajectories were collected for each path, resulting in forty trajectories. Each path included two turns, providing a robust test for the algorithm under various motion scenarios. Details of the sampling paths are presented in Table 2.

**Figure 14:**
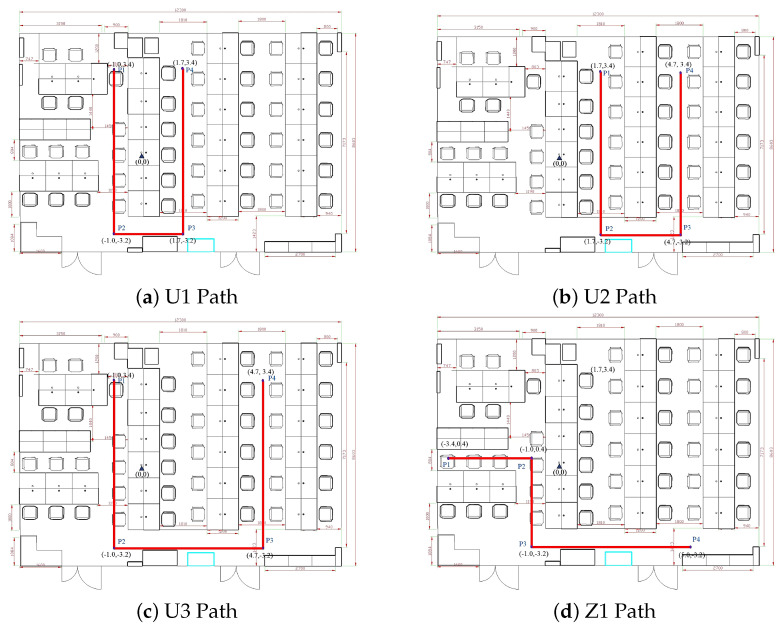
Schematic diagram of sampling paths: (**a**) U1 path; (**b**) U2 path; (**c**) U3 path; (**d**) Z1 path. The diagrams illustrate the layout and trajectory of each sampling path used in the experiment.

The schematic diagrams of the four sampling paths on the map are shown in Figure 14.

#### 4.1.1. Pedestrian Dead Reckoning Performance

The performance of the proposed pedestrian dead reckoning (PDR) algorithm was evaluated through comparative experiments. The following methods were experimented with for comparison:

- FIN-KNN: A wireless signal fingerprinting method that uses the K-Nearest Neighbors (KNN) algorithm to estimate the user’s position. This is one of the most widely used fingerprinting-based positioning algorithms.
- PDR-Q: A pedestrian dead reckoning algorithm that establishes a pedestrian step model, employing step frequency detection, step length estimation, and heading estimation in three steps to derive the pedestrian’s position. The heading estimation in this method uses the traditional quaternion update approach.
- PDR-A: A pedestrian dead reckoning algorithm that establishes a pedestrian step model and uses step frequency detection, step length estimation, and heading estimation in three steps. However, this method applies the newly proposed angular displacement projection method for heading estimation.

To assess the performance of these methods, three metrics were used:

- Average localization error (ALE): The mean physical distance between the predicted and actual positions across all test points.
- Cumulative distribution function (CDF): A curve representing the cumulative distribution of positioning errors. The horizontal axis represents the positioning error, and the vertical axis represents the proportion of test points with errors below the given value.
- Percentile localization error: On the CDF curve, the error corresponds to a specific percentage on the vertical axis (e.g., 50%, 75%, or 90%). These characterize the error range for a given proportion of test points.

**Figure 15:**
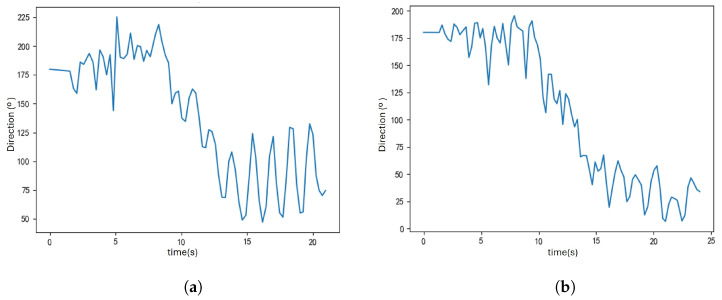
This figure illustrates the heading estimation results using the PDR-Q algorithm. It shows the waveform of the pedestrian’s heading changes along the U1 and U2 trajectories. (**a**) Heading estimation for the U1 trajectory. (**b**) Heading estimation for the U2 trajectory.

**Figure 16:**
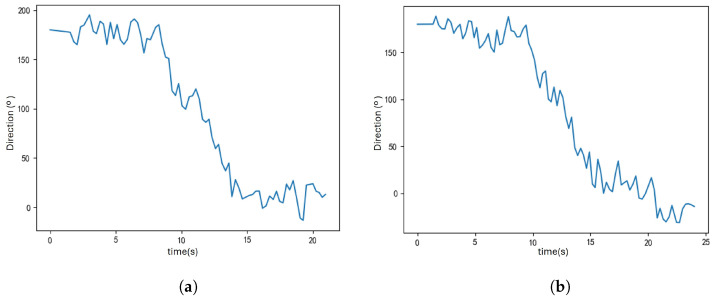
This figure illustrates the heading estimation results using the PDR-A algorithm, showing the waveform of the pedestrian’s heading changes along the U1 trajectory. (**a**) heading estimation for the U1 trajectory, (**b**) Heading estimation for the U2 trajectory.

The main difference between PDR-Q and PDR-A lies in the heading estimation algorithm. To compare the effectiveness of these two methods, their heading estimation performance was observed on two trajectories from the U1 and U2 paths. Both trajectories included a U-shaped turn, where the pedestrian’s heading changed from 180° to 0°. The results of the heading estimation are shown in Figure 15 and Figure 16.

The heading estimation results indicate that PDR-Q and PDR-A can capture changes in the pedestrian’s heading. However, the PDR-A method is more precise and fluctuates less, while the PDR-Q method exhibits more significant variability. Because the PDR-A waveform is more stable, it is better suited for the turn recognition process, allowing for more accurate turn detection and reducing false detections.

**Figure 17:**
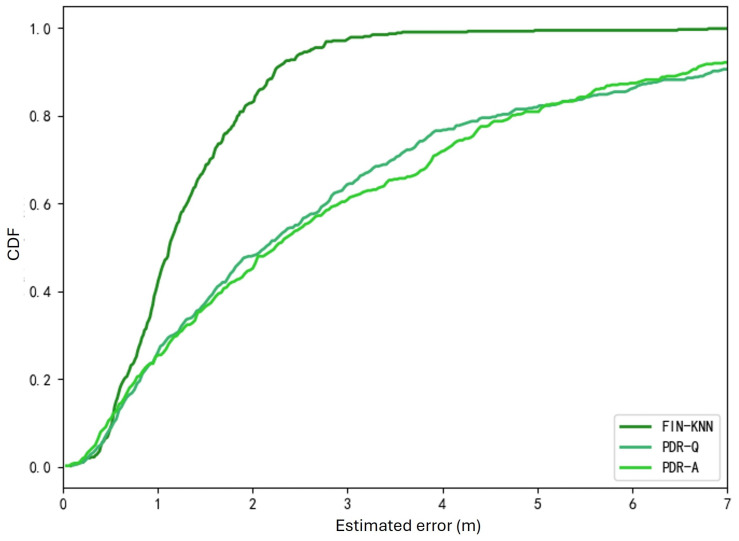
Cumulative distribution function (CDF) of errors for U1 path.

The positioning performances of the two algorithms were tested on 40 trajectories, with the results shown in Table 3 and Figure 17. The positioning accuracy of the PDR-A algorithm is very close to that of the PDR-Q algorithm, with PDR-A achieving a 0.06% higher accuracy.

However, both PDR-based positioning algorithms exhibit significantly lower accuracy than the wireless signal fingerprinting method. While PDR algorithms can precisely track a target’s trajectory over short distances, the error accumulates as the walking distance increases. These results further demonstrate that PDR algorithms alone are unsuitable for standalone positioning.

In the CDF curves shown in Figure 17, the positioning performance of the PDR-Q and PDR-A algorithms is also close, with PDR-A performing slightly better. However, the wireless signal fingerprinting algorithm outperforms both PDR algorithms, which is determined by the inherent nature of the positioning sources.

The PDR-Q method involves more complex calculations than the PDR-A method. Computation times were measured to evaluate the computational complexities of the two algorithms. The experiments were conducted on a computer with an Intel (R) Core (TM) i7-4790 CPU @ 3.60 GHz and 16 GB of RAM (Intel, Santa Clara, CA, USA). The computation times for the two algorithms on different numbers of trajectories are shown in Table 4.

The experimental results align with theoretical expectations, showing that PDR-A requires less computation time and involves fewer calculations than PDR-Q.

PDR-A outperforms PDR-Q regarding heading stability, positioning error, and computation time among the two PDR algorithms tested in this chapter. However, the positioning results of both algorithms still show a particular gap compared to the results achieved using the wireless signal fingerprinting method.

#### 4.1.2. Fusion Positioning Algorithm Performance

The performance of the proposed fusion positioning algorithm was evaluated through comparative experiments. The following methods were compared:

- FIN-KNN: A wireless signal fingerprinting algorithm using the K-nearest neighbors (KNN) method to determine the user’s position. This is a commonly used fingerprinting-based positioning approach.
- PDR-Q: The PDR algorithm introduced in Section 3 constructs a pedestrian step model and calculates the position using three steps: step frequency detection, step length estimation, and heading estimation. Heading estimation is based on the traditional quaternion update method.
- PDR-A: The PDR algorithm introduced in Section 3 also constructs a pedestrian step model and calculates the position using the same three steps. However, this method employs the newly proposed angular displacement projection method for heading estimation.
- MIX-7: The fusion positioning algorithm introduced in Section 4 combines PDR and wireless fingerprinting results to calculate the pedestrian’s position. This method uses a sliding window size of 7 for turn detection.
- MIX-9: A variation of the fusion positioning algorithm introduced in Section 3, using a sliding window size of 9 for turn detection while keeping all other parameters identical to MIX-7.

In the comparative schemes, FIN-KNN relies solely on wireless signals, while PDR-Q and PDR-A are based exclusively on inertial sensors, representing single-source methods. MIX-7 and MIX-9 are multi-source fusion algorithms. To ensure fairness, the same methods and parameters are used for step frequency detection and step length estimation in PDR-Q and PDR-A, differing only in their heading estimation approaches. Similarly, MIX-7 and MIX-9 differ only in their sliding window sizes, with all other parameters kept the same.

The experiments were conducted on 40 trajectories based on paths U1, U2, U3, and Z1. Each trajectory included two turns with different turning directions. These complex trajectory scenarios were used to validate the effectiveness of the proposed algorithm.

The positioning performance evaluation metrics are consistent with Section 4.1.2, including:

##### U1 Path

**Figure 18:**
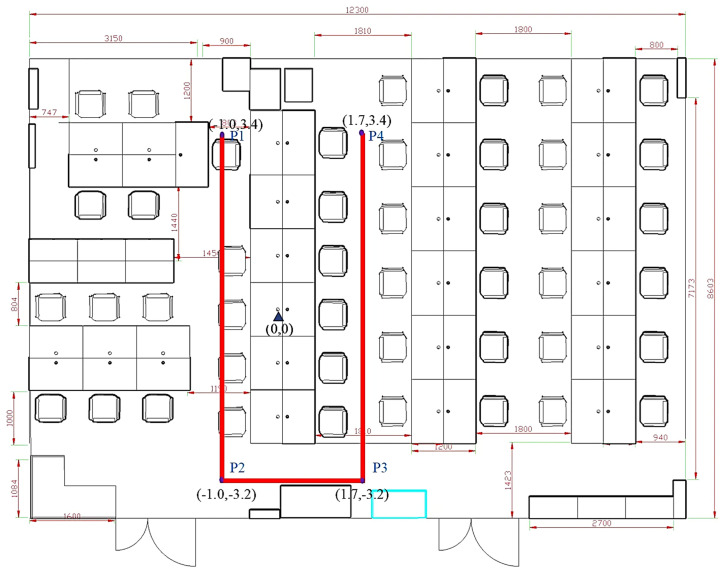
Schematic diagram of the U1 trajectory.

The U1 path, as shown in Figure 18, consists of the following:

- Forward trajectory: P1 → P2 → P3 → P4.
- Reverse trajectory: P4 → P3 → P2 → P1.

**Figure 19:**
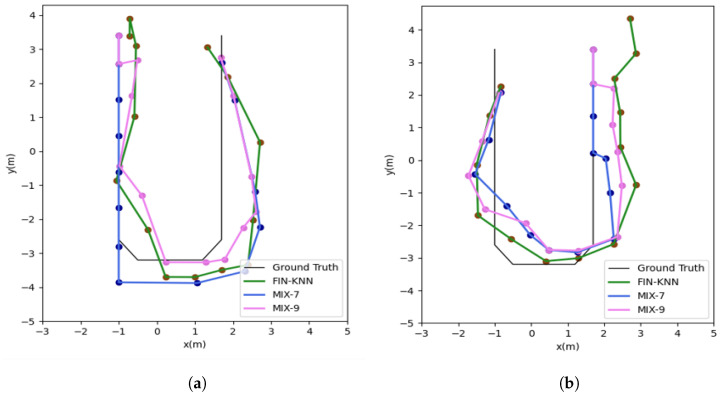
This figure shows both the forward and reverse positioning trajectories for the U1 path, illustrating the sequences P1 → P2 → P3 → P4 and P4 → P3 → P2 → P1, respectively. (**a**) U1 forward positioning trajectory. (**b**) U1 reverse positioning trajectory.

The positioning trajectories for the U1 path are shown in Figure 19.

**Figure 20:**
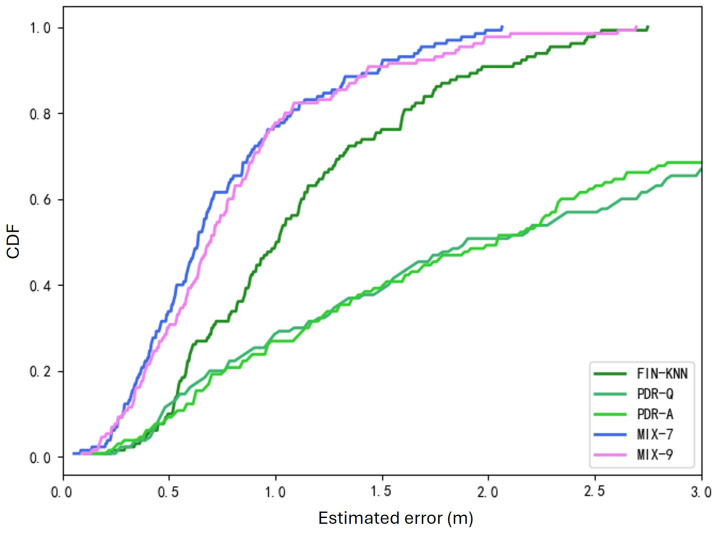
Cumulative distribution function (CDF) of errors for U1 path.

The two trajectories in Figure 19 demonstrate that the proposed MIX-7 and MIX-9 algorithms achieve better trajectory tracking performance. Table 5 and Figure 20 present the quantitative error analysis for the U1 path.

The proposed MIX-7 method achieves the best positioning results among the various comparison algorithms. Its positioning accuracy is 67.2% higher than the best PDR method (PDR-A) and 32.8% higher than the wireless fingerprinting method (FIN-KNN). This demonstrates that the multi-source fusion positioning approach can achieve optimal results on the U1 U-shaped trajectory.

##### U2 Path

**Figure 21:**
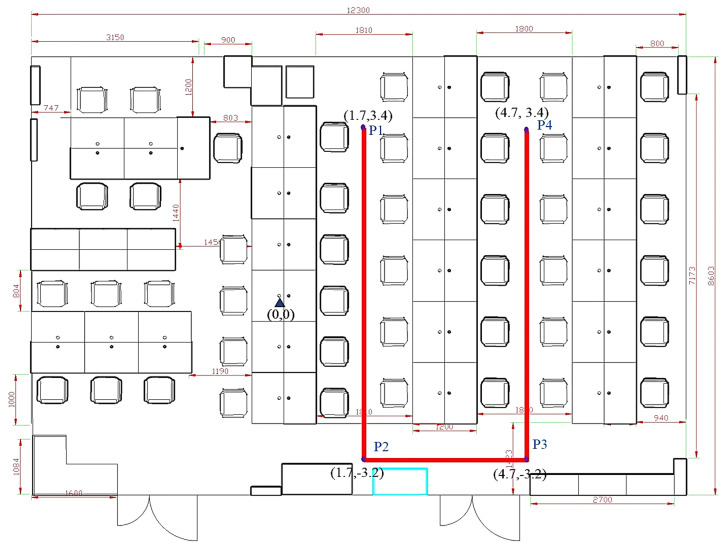
Schematic diagram of the U2 trajectory.

Figure 21 shows the U2 path. The forward trajectory is collected in the sequence P1 → P2 → P3 → P4, while the reverse trajectory is collected in the sequence P4 → P3 → P2 → P1.

**Figure 22:**
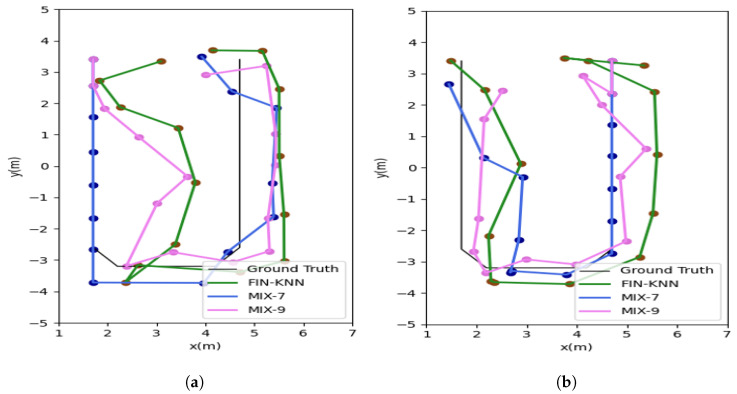
Positioning trajectories for the U2 path. (**a**) U2 forward positioning trajectory. (**b**) U2 reverse positioning trajectory.

The positioning trajectories for the U2 path are shown in Figure 22.

**Figure 23:**
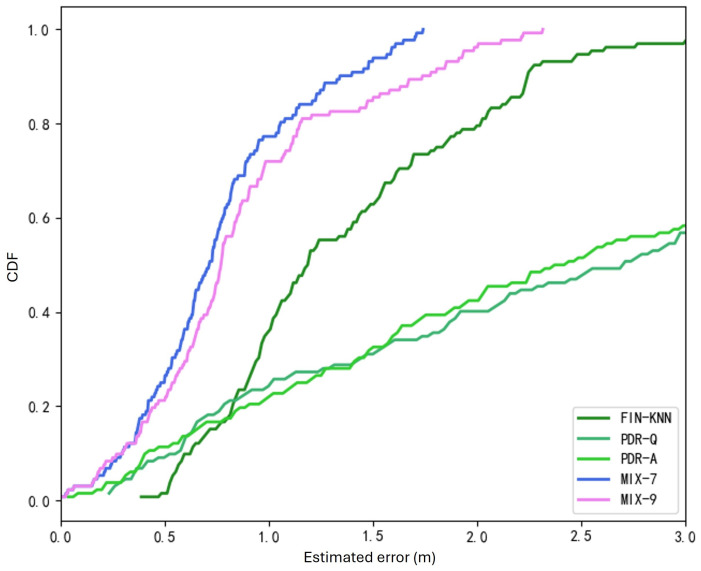
Cumulative distribution function (CDF) of errors for U2 path.

The two trajectories in Figure 22 demonstrate that the proposed MIX-7 and MIX-9 algorithms achieve better trajectory-tracking performance. Table 6 and Figure 23 present the quantitative error analysis for the U2 path.

The proposed MIX-7 method achieves the best positioning results among the various comparison algorithms. Its positioning accuracy is 74.5% higher than that of the best PDR method (PDR-A) and 47.5% higher than that of the wireless fingerprinting method (FIN-KNN). This demonstrates that the multi-source fusion positioning approach can achieve optimal results on the U2 U-shaped trajectory.

##### The 10 Trajectories of the U3 Path

**Figure 24:**
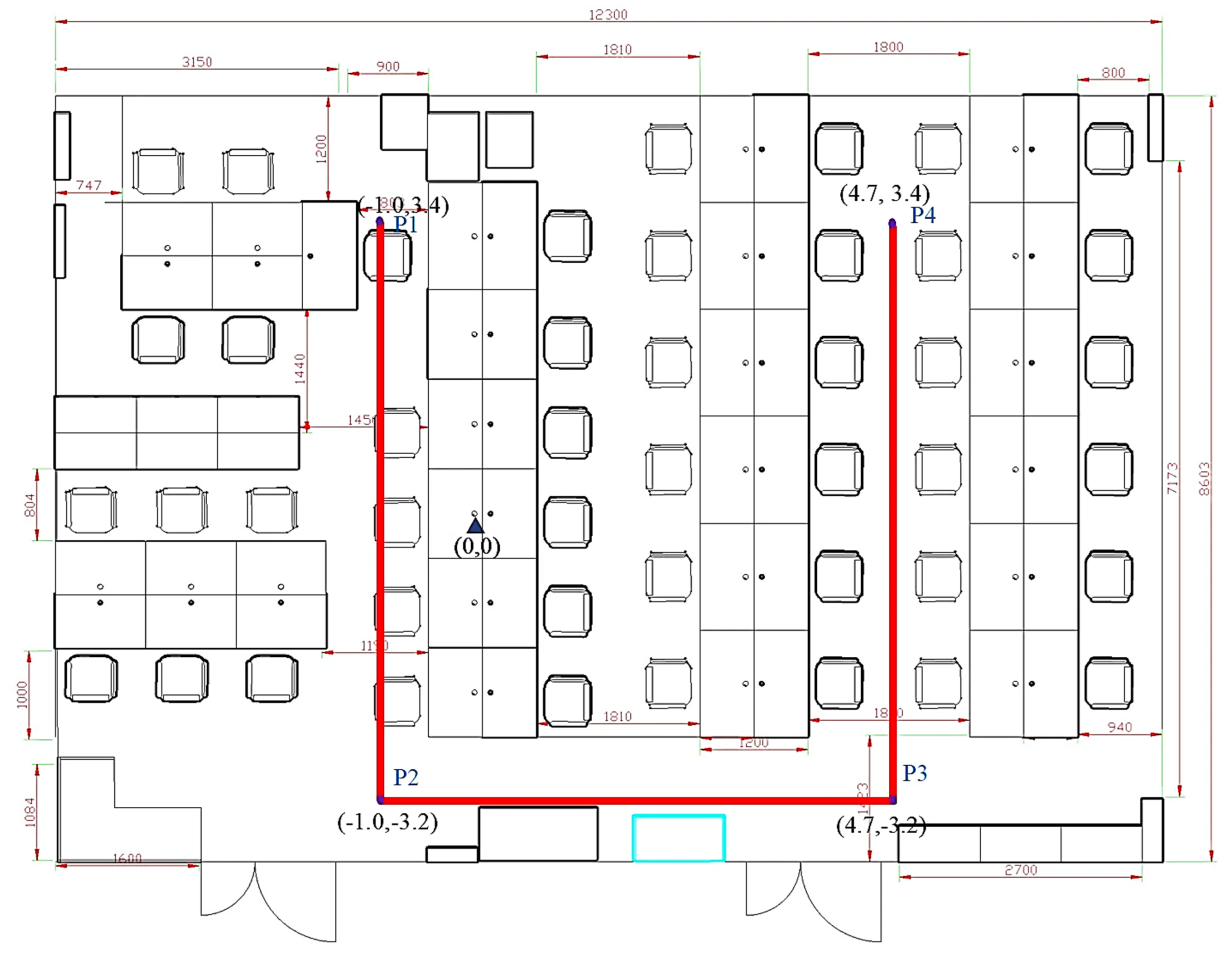
Schematic diagram of the U3 trajectory.

**Figure 25:**
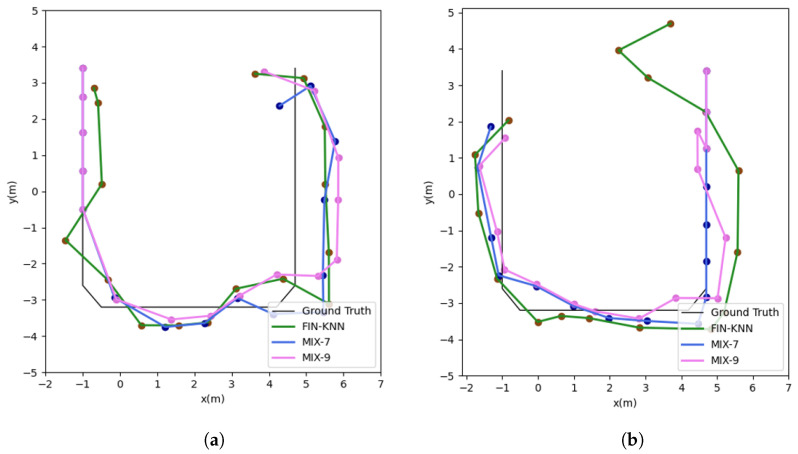
Positioning trajectories for the U3 path. (**a**) U3 forward positioning trajectory. (**b**) U3 reverse positioning trajectory.

**Figure 26:**
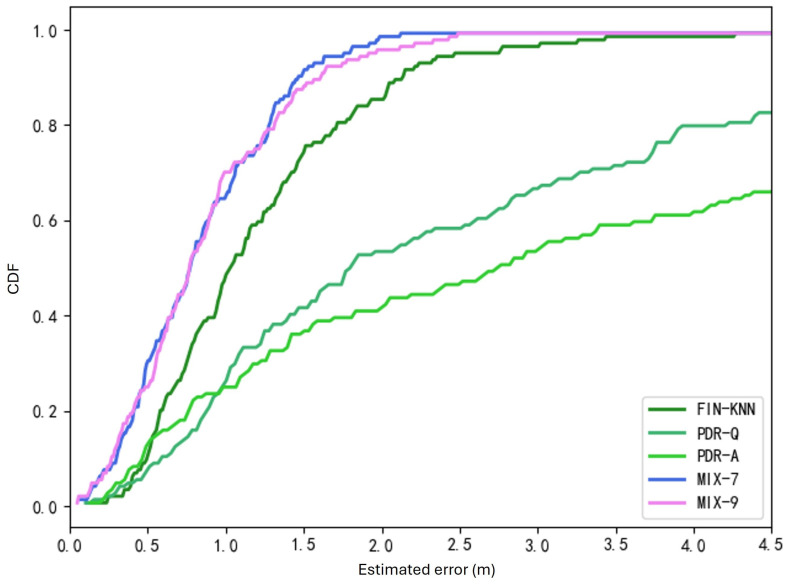
Cumulative distribution function (CDF) of errors for U3 path.

Figure 24 shows the U3 path. The forward trajectory is collected in the sequence P1 → P2 → P3 → P4, while the reverse trajectory is collected in the sequence P4 → P3 → P2 → P1. Figure 25 shows the U3 positioning trajectories are depicted for both forward and reverse paths. Table 7 presents various methods’ average and percentile errors. Figure 26 illustrates the cumulative distribution function (CDF) of positioning errors, offering a comparative analysis of algorithm performance.

The proposed MIX-7 method achieves the best positioning results among the various comparison algorithms. Its positioning accuracy is 68.2% higher than that of the best PDR method (PDR-Q) and 30.1% higher than that of the wireless fingerprinting method (FIN-KNN). This demonstrates that the multi-source fusion positioning approach can achieve optimal results on the U3 U-shaped trajectory.

##### Z1 Path

**Figure 27:**
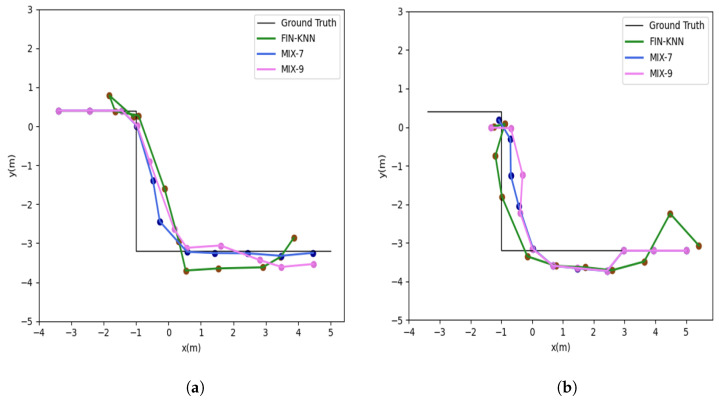
Positioning trajectories for the Z1 Path. (**a**) Z1 forward positioning trajectory. (**b**) Z1 reverse positioning trajectory.

**Figure 28:**
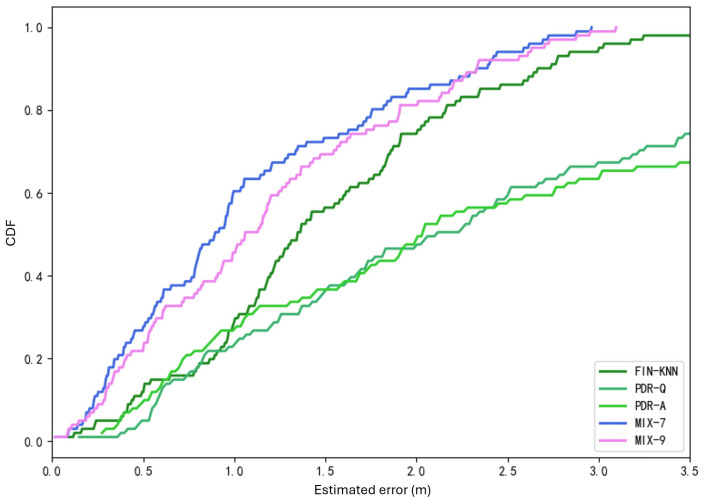
Cumulative distribution function (CDF) of errors for Z1 path.

**Figure 29:**
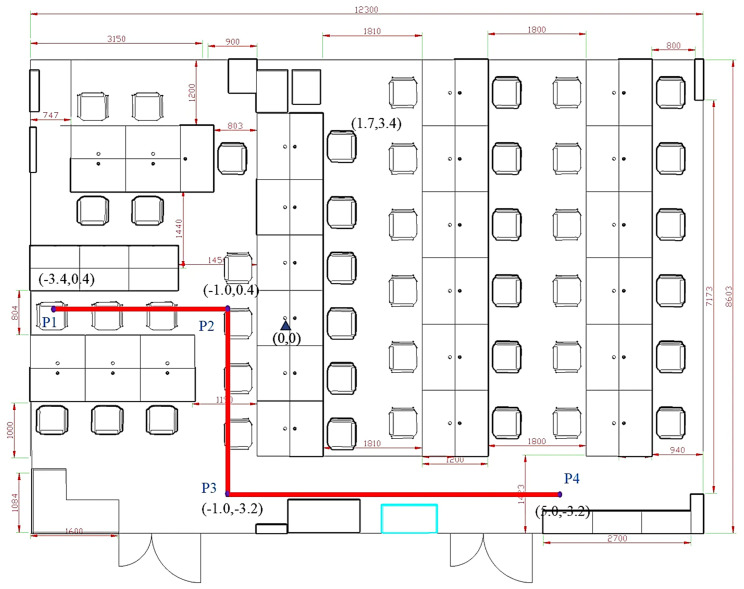
The Z1 path trajectory showing the forward sequence P1 → P2 → P3 → P4 and the reverse sequence P4 → P3 → P2 → P1.

The positioning trajectories for the Z1 path are shown in Figure 27. The two trajectories in Figure 27 demonstrate that the proposed MIX-7 and MIX-9 algorithms achieve better trajectory-tracking performance. Table 8 and Figure 28 present the quantitative error analysis for the Z1 path. Figure 29 illustrates the Z1 path, capturing both forward and reverse trajectories. The forward trajectory follows a sequential path P1 → P2 → P3 → P4, ensuring a structured representation of movement. Conversely, the reverse trajectory retraces the path in the opposite order, progressing from P4 → P3 → P2 → P1. This bidirectional data collection provides a comprehensive view of movement dynamics along the Z1 path. 

**Figure 30:**
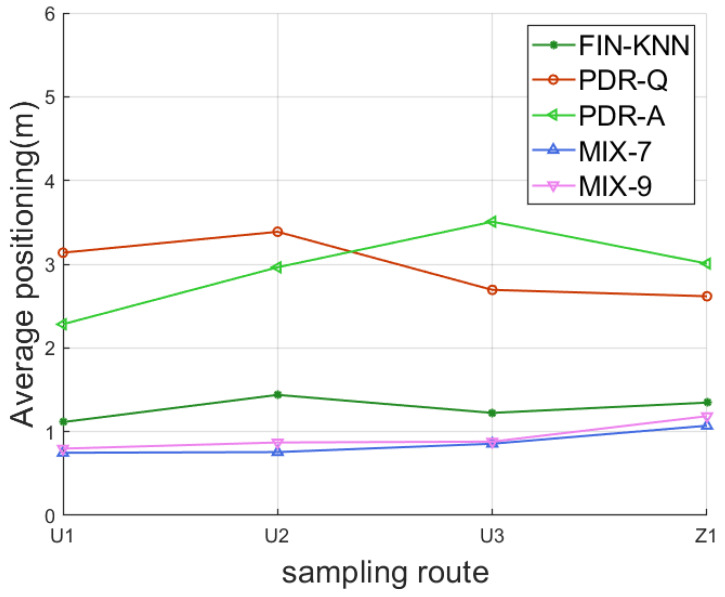
Average error curves for each sampling path.

The proposed MIX-7 method achieves the best positioning results among the various comparison algorithms. Its positioning accuracy is 59.1% higher than that of the best PDR method (PDR-Q) and 20.5% higher than that of the wireless fingerprinting method (FIN-KNN). This demonstrates that the multi-source fusion positioning approach can achieve optimal results on the Z1 Z-shaped trajectory. Table 9 and Figure 30 show the positioning error evaluations for each algorithm across all trajectories. The results indicate that the proposed MIX-7 algorithm achieves the best positioning accuracy across all paths, fully demonstrating the effectiveness and robustness of the algorithm presented in this chapter.

**Figure 31:**
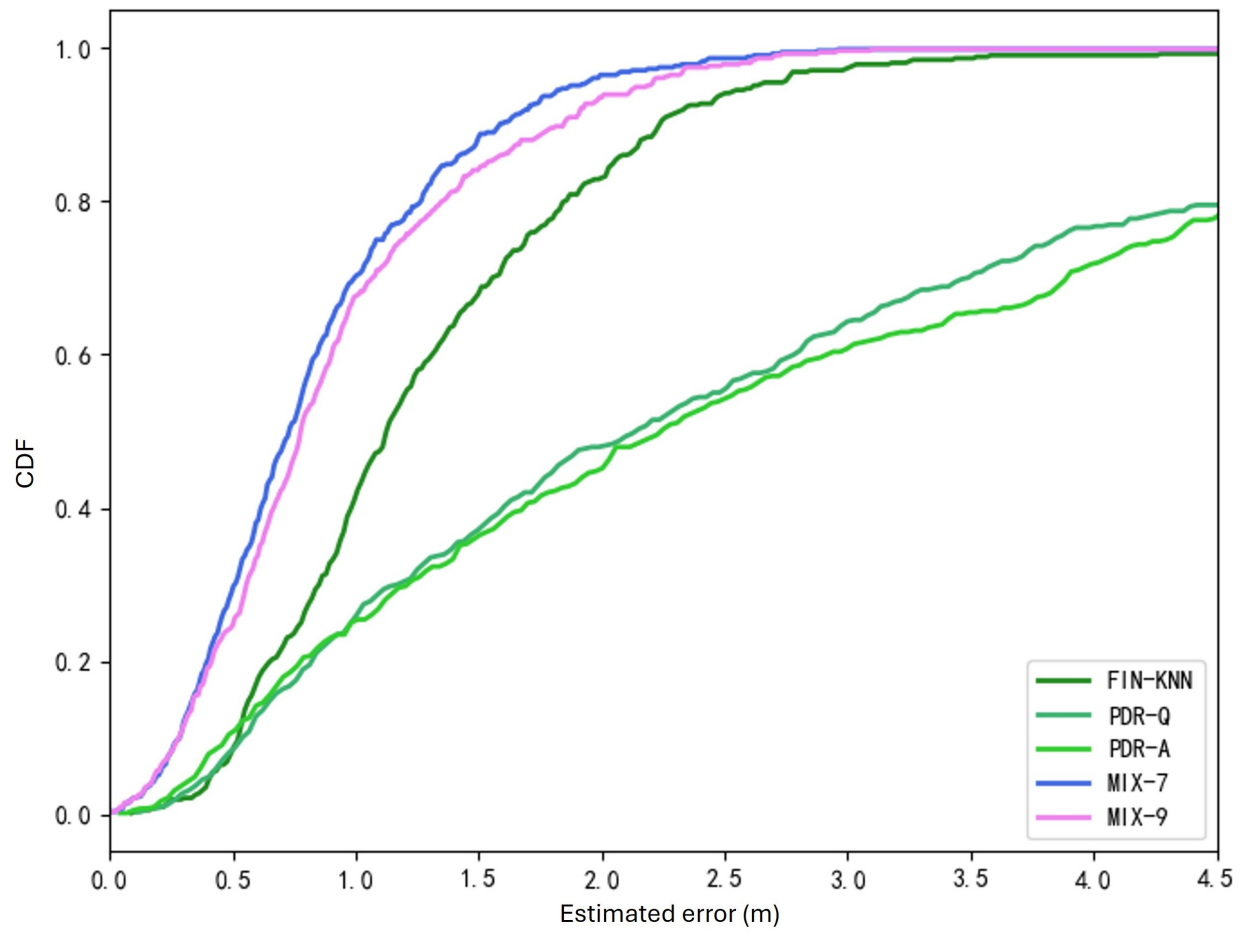
Overall CDF of positioning errors (40 trajectories).

Table 10 and Figure 31 present the overall positioning error statistics for all 40 trajectories. The conclusion is consistent with each path’s conclusions: The fusion positioning algorithm based on inertial sensors and signal fingerprinting proposed in this chapter achieves the best positioning accuracy. It improves positioning accuracy by 35.3% compared to direct wireless signal positioning and by 71.4% compared to the PDR-A method, demonstrating excellent positioning performance.

## 5. Discussion

The experimental results demonstrate the effectiveness of the proposed fusion positioning algorithm based on inertial sensors and signal fingerprints. This discussion section examines the implications of these findings, highlights limitations, and suggests future research directions.

### 5.1. Implications of the Proposed Algorithm

The proposed algorithm significantly improves indoor positioning accuracy by addressing the limitations of single-source methods. The following key aspects underline its impact:

#### 5.1.1. Enhanced Accuracy and Robustness

By integrating pedestrian dead reckoning (PDR) with wireless signal fingerprinting, the algorithm leverages the complementary strengths of both approaches. PDR provides continuous trajectory tracking, while signal fingerprinting corrects cumulative errors:

- The weighted fusion approach enables real-time adaptation to dynamic environments, achieving an average localization error (ALE) improvement of up to 35.3% over FIN-KNN and 71.4% over PDR-A.
- Cumulative distribution function (CDF) analysis significantly reduces significant errors, particularly in scenarios with frequent turns and obstacles.

#### 5.1.2. Computational Efficiency

Despite integrating two distinct methods, the proposed algorithm demonstrates competitive computational efficiency. The processing time is slightly lower than that of PDR-Q and PDR-A, making it feasible for real-time applications in resource-constrained environments. This advantage arises from the efficient implementation of motion state recognition and the fusion framework.

#### 5.1.3. Trajectory Tracking in Complex Environments

The experimental results show that the algorithm closely tracks actual trajectories, outperforming single-source methods, particularly in U-shaped and Z-shaped paths. This capability makes the algorithm suitable for complex indoor scenarios like warehouses, airports, and shopping malls.

### 5.2. Limitations of the Current Approach

While the proposed algorithm achieves significant improvements, it is not without limitations:

- Dependence on signal quality: The algorithm’s performance relies on the quality and availability of wireless signal fingerprints. In environments with highly variable or weak signals, positioning accuracy may degrade.
- Cumulative errors in extended use: Although the fusion mitigates PDR’s cumulative errors, residual drift may persist in prolonged trajectories, particularly if signal fingerprint corrections are infrequent.
- Scalability challenges: The experimental setup was conducted in a laboratory environment with controlled conditions. Scalability to larger and more dynamic environments requires further validation.

#### 5.2.1. Time Complexity of FIN-KNN

The FIN-KNN algorithm, based on the K-nearest neighbors (KNN) algorithm, has a time complexity of O(n·d), where *n* is the number of samples in the dataset, and *d* is the dimensionality of the feature space. In the context of this study, *n* represents the number of Wi-Fi signal fingerprints, and *d* corresponds to the RSSI features collected from multiple access points.

While FIN-KNN is effective for indoor localization, the computational requirements scale with the dataset size, potentially increasing runtime for large environments. This limitation makes FIN-KNN less suitable for real-time applications, particularly in scenarios with dynamic signal variations or high-density environments. The proposed MIX-7 and MIX-9 algorithms, with their linear time complexity and fusion-based approach, provide improved computational efficiency and scalability, making them better suited for such applications.

#### 5.2.2. Practical Implications of the Results

The experimental findings validate the proposed fusion positioning algorithm as a robust solution for dynamic and complex indoor environments. These results have practical implications across multiple domains:

- Navigation systems: The algorithm’s enhanced accuracy and trajectory-tracking capabilities make it suitable for indoor navigation in environments such as airports, shopping malls, and hospitals. Mitigating cumulative errors in extended paths ensures reliable guidance even in non-linear trajectories and dynamic obstacle conditions.
- Robotics and automation: The algorithm’s ability to adapt dynamically to motion state changes makes it a viable solution for robotic applications, including automated warehouse systems and delivery robots. Its computational efficiency further ensures real-time performance in resource-constrained environments with frequent trajectory adjustments.
- Asset tracking and management: The algorithm effectively tracks high-value assets in industrial and healthcare settings by leveraging existing Wi-Fi infrastructure. Its adaptability ensures robust performance in environments with signal variability or frequent reconfigurations.
- Dynamic indoor environments: The experimental results highlight the algorithm’s ability to manage diverse motion scenarios, including U-shaped and Z-shaped paths, making it particularly suitable for environments with high mobility or frequent layout changes.
- Scalability to real-world deployments: Integrating efficient fusion and adaptive strategies reduces deployment costs and enhances scalability, enabling seamless integration with existing infrastructure for large-scale implementations.

### 5.3. Future Research Directions

To address the identified limitations and extend the applicability of the proposed algorithm, future research should focus on the following areas:

- Dynamic signal adaptation: Future work should aim to develop algorithms capable of adapting to real-time signal quality variations. Advanced filtering techniques or machine learning-based corrections could be explored to adjust to environmental changes dynamically, ensuring consistent positioning accuracy.
- Error minimization for long trajectories: Long trajectories often result in a cumulative drift that degrades positioning accuracy. Hybrid approaches incorporating map-matching techniques or additional sensor modalities, such as geomagnetic sensors or visual odometry, should be investigated to mitigate these errors effectively.
- Validation in real-world scenarios: Large-scale experiments in diverse and challenging environments, such as multi-level buildings, crowded spaces, and industrial settings, are essential for validating the algorithm’s robustness and scalability. These experiments would provide insights into its adaptability in dynamic and complex scenarios.
- Energy efficiency: Optimizing the algorithm for low power consumption is critical for enabling its deployment on wearable and mobile devices. Future research should explore lightweight computational frameworks that reduce energy usage, allowing long-term, continuous use in real-world applications.
- Integration with emerging technologies: Future work could explore its integration with emerging technologies such as 6G communication networks, IoT-enabled devices, and edge computing platforms to enhance the algorithm’s applicability further. These advancements could improve localization accuracy and enable seamless scalability for larger environments.

By addressing these directions, future research can overcome current limitations and pave the way for more robust, scalable, and energy-efficient indoor localization systems.

### 5.4. Broader Implications

The proposed algorithm demonstrates significant potential for practical applications in various domains:

- Navigation systems: The enhanced accuracy and robustness make it suitable for indoor navigation in airports, shopping malls, and hospitals.
- Industrial automation: Its ability to handle complex trajectories is an ideal solution for robotic navigation and asset tracking in warehouses.
- Augmented reality (AR): The real-time positioning capabilities can support AR systems that require precise spatial awareness in indoor environments.

Overall, the fusion positioning algorithm effectively bridges the gap between accuracy and computational efficiency, offering a viable solution for indoor localization. By addressing current limitations and exploring future enhancements, the algorithm has the potential to become a cornerstone in next-generation positioning systems.

## 6. Conclusions

This study proposed and experimentally evaluated two fusion positioning algorithms, MIX-7 and MIX-9, for indoor localization by integrating inertial sensor data with wireless signal fingerprinting. The experiments demonstrated that MIX-7 achieved the best overall performance, with an average localization error of 0.85 m, while MIX-9 provided a comparable accuracy of 0.92 m. Standalone methods were also analyzed, including pedestrian dead reckoning with quaternion updates (PDR-Q) and angular displacement projection (PDR-A). The fusion algorithms significantly outperformed these methods, with MIX-7 achieving a 71.4% reduction in error compared to PDR-A and a 35.3% improvement over FIN-KNN. These results underscore the ability of MIX-7 and MIX-9 to effectively handle cumulative errors inherent in PDR methods and mitigate signal variability challenges in wireless-only systems. Based on received signal strength indicators (RSSIs), Wi-Fi signals were employed for fingerprinting, ensuring compatibility with existing infrastructure and practical deployment. Integrating inertial sensor data and Wi-Fi fingerprinting improved localization accuracy and robustness in dynamic indoor environments. Supplementary computational analysis: The experiments included computational time measurements for PDR-Q and PDR-A. PDR-Q required 7.81 s to process 40 trajectories, while PDR-A required 7.26 s, highlighting PDR-A’s computational efficiency due to angular displacement projection. Although computational times for MIX-7 and MIX-9 were not explicitly measured, their sliding window sizes suggest slightly higher computational demands for MIX-9, balancing this overhead with improved robustness in dynamic scenarios. This study highlights the potential of MIX-7 and MIX-9 as accurate, scalable, and efficient solutions for indoor localization in navigation, robotics, and asset-tracking applications. Future research will focus on integrating additional signal sources, such as Bluetooth or ultra-wideband (UWB), to further enhance localization accuracy and robustness in complex environments.

## Figures and Tables

**Table 1:** Summary of related works on indoor positioning systems.

Reference	Contribution	Technique Used	Limitations
Qian et al. [2]	Strong tracking particle filter for improved indoor positioning	Particle filter, Chi-square test	High computational cost limits real-time applications; sensitive to dynamic conditions
Song et al. [3]	Multi-node fusion using wearable inertial sensors	Learning-based fusion	Scalability issues and lack of adaptability to dynamic environments
Wang et al. [5]	Fusion positioning using Wi-Fi, PDR, and geomagnetic fields	Improved particle filter	Sensitive to environmental interference; requires frequent signal updates
Cheng et al. [14]	Implicit unscented particle filter for indoor fusion positioning	Unscented particle filter	Computational overhead makes it unsuitable for real-time applications
Hua et al. [11]	BLE-IMU fusion positioning for Industry 4.0	Deep learning- based fusion	High computational cost, dependency on large training datasets
Yu et al. [22]	High-precision multi-feature fusion for inertial sensor networks	Multi-feature fusion	Limited generalizability to large-scale and multi-device environments
Huang et al. [18]	WiFi and IMU fusion using swarm optimization	Swarm optimization	Environmentally sensitive, requires significant parameter tuning
Wang et al. [32]	Robust sensor fusion for indoor wireless localization	Robust sensor fusion	Scalability challenges and reduced performance in dynamic environments
Sun et al. [20]	UWB/Inertial fusion localization using convolutional neural networks	Convolutional neural networks	Costly infrastructure setup, limited scalability for large environments
Tang et al. [13]	Wi-Fi RSSI-based data augmentation for large-scale localization	Multi-output Gaussian process	Computational costs in large deployments; signal fluctuations affect stability
Milano et al. [15]	BLE-based indoor localization performance enhancement	BLE fingerprinting, signal modeling	Limited to BLE hardware; susceptible to environmental obstructions
Ciuffreda et al. [17]	Multi-sensor fusion with PIR and ultrasonic sensors for people tracking	PIR-Ultrasonic sensor fusion	High dependency on direct line-of-sight; signal interference reduces accuracy
Senel et al. [31]	Multi-object tracking using real-time sensor fusion	Multi-sensor fusion	Increased computational demand; sensor misalignment affects accuracy
Choi et al. [33]	Sensor fusion of LiDAR and infrared cameras for positioning	LiDAR-Infrared fusion	High infrastructure cost; sensitive to lighting conditions
Hu and Assaad et al. [25]	Digital twin framework for real-time indoor positioning	LiDAR-3D mapping, IoT sensing	High data processing requirements; computationally intensive
Alvarez-Merino et al. [7]	Evaluation of 5G, Wi-Fi, and sensor fusion for localization	Multi-signal fusion, 5G RSSI-based localization	Incomplete maps affect accuracy; computational overhead in 5G processing

**Table 2:** Details of the sampling paths. The table lists each sampling path’s number of trajectories, path length, steps, and turns.

Path Name	Number of Trajectories	Path Length (m)	Number of Steps	Number of Turns
U1	10	15.9	15	2
U2	10	16.2	15	2
U3	10	18.9	17	2
Z1	10	12.0	13	2

**Table 3:** Average error and percentile error for each method (unit: meters).

Algorithm	Average Error	50% Error	75% Error	90% Error
FIN-KNN	1.307	1.122	1.699	2.241
PDR-Q	2.972	2.141	3.822	6.883
PDR-A	2.952	2.249	4.298	6.580

**Table 4:** Computation time for each method (unit: seconds).

Algorithm	10 Trajectories	20 Trajectories	30 Trajectories	40 Trajectories
PDR-Q	1.98	3.89	6.40	7.81
PDR-A	1.88	3.59	5.73	7.26

**Table 5:** Error analysis of U1 path for each method (unit: meters).

Algorithm	Average Error	50% Error	75% Error	90% Error
FIN-KNN	1.114	1.007	1.482	1.952
PDR-Q	3.137	1.912	4.572	7.252
PDR-A	2.283	2.016	3.640	4.263
MIX-7	0.748	0.640	0.962	1.487
MIX-9	0.797	0.695	0.960	1.434

**Table 6:** U2 path error comparison for each method (unit: meters).

Algorithm	Average Error	50% Error	75% Error	90% Error
FIN-KNN	1.439	1.170	1.820	2.258
PDR-Q	3.387	2.646	4.119	8.752
PDR-A	2.963	2.418	4.279	6.173
MIX-7	0.756	0.711	0.948	1.340
MIX-9	0.871	0.770	1.103	1.734

**Table 7:** Positioning accuracy evaluation for U3 path trajectories (unit: meters).

Algorithm	Average Error	50% Error	75% Error	90% Error
FIN-KNN	1.224	1.032	1.521	2.125
PDR-Q	2.693	1.790	3.718	5.896
PDR-A	3.507	2.746	5.524	7.622
MIX-7	0.856	0.769	1.194	1.461
MIX-9	0.881	0.764	1.195	1.593

**Table 8:** Quantitative error analysis for Z1 Path (unit: meters).

Algorithm	Average Error	50% Error	75% Error	90% Error
FIN-KNN	1.347	1.521	2.013	2.663
PDR-Q	2.617	2.112	3.510	6.192
PDR-A	3.007	1.995	4.779	7.622
MIX-7	1.071	0.889	1.626	2.338
MIX-9	1.185	1.055	1.731	2.333

**Table 9:** Average error for each path (unit: meters).

Algorithm	U1	U2	U3	Z1
FIN-KNN	1.114	1.439	1.224	1.347
PDR-Q	3.137	3.387	2.693	2.617
PDR-A	2.283	2.963	3.507	3.007
MIX-7	0.748	0.756	0.856	1.071
MIX-9	0.797	0.871	0.881	1.185

**Table 10:** Average error and percentile error for each method (unit: meters).

Algorithm	Average Error	50% Error	75% Error	90% Error
FIN-KNN	1.307	1.122	1.699	2.241
PDR-Q	2.972	2.141	3.822	6.883
PDR-A	2.952	2.249	4.298	6.580
MIX-7	0.845	0.728	1.111	1.601
MIX-9	0.918	0.777	1.191	1.832

## Data Availability

No new data were created or analyzed in this study. Data sharing is not applicable to this article.

## Abbreviations

IPS	indoor positioning system
RSSI	received signal strength indicator
UWB	ultra-wideband
IMU	inertial measurement unit
PDR	pedestrian dead reckoning
PDR-Q	pedestrian dead reckoning with quaternion update
PDR-A	pedestrian dead reckoning with angular displacement projection
BLE	Bluetooth low energy
MIX-7	fusion algorithm with sliding window size 7
MIX-9	fusion algorithm with sliding window size 9
ALE	average localization error
CDF	cumulative distribution function
FIN-KNN	fingerprint indoor navigation using K-nearest neighbors
